# Gastrodin Attenuates Bilateral Common Carotid Artery Occlusion-Induced Cognitive Deficits via Regulating Aβ-Related Proteins and Reducing Autophagy and Apoptosis in Rats

**DOI:** 10.3389/fphar.2018.00405

**Published:** 2018-04-26

**Authors:** Bo Liu, Jian-Mei Gao, Fei Li, Qi-Hai Gong, Jing-Shan Shi

**Affiliations:** ^1^School of Pharmacy, Shanghai University of Traditional Chinese Medicine, Shanghai, China; ^2^Key Laboratory of Basic Pharmacology of Ministry of Education and Joint International Research Laboratory of Ethnomedicine of Ministry of Education, Zunyi Medical University, Zunyi, China

**Keywords:** gastrodin, cognitive deficits, hippocampus, Aβ-related proteins, autophagy, apoptosis

## Abstract

Gastrodin (GAS), an active constituent extracted from *Gastrodia elata* Blume, is used to treat ischemic stroke, epilepsy, dizziness, and dementia for centuries in China. This study examined its effects on vascular dementia (VD) and the underlying molecular mechanisms. VD was established by ligation of bilateral common carotid artery occlusion (BCCAO). A total of 7 days after BCCAO surgery, GAS (15, 30, and 60 mg/kg) was orally administered for 28 consecutive days to evaluate therapeutic effects. Cognitive function was tested by the Morris water maze. The neuronal morphological changes were examined via Hematoxylin–Eosin staining. Flow cytometry was used for evaluating apoptosis in the hippocampi. The target protein expression was examined by Western blot. The results showed that BCCAO induced cognitive impairment, hippocampus CA1 and CA3 pyramidal neuron damage, beta-amyloid (Aβ) deposition, excessive autophagy, and apoptosis. GAS treatment significantly improved BCCAO-induced cognitive deficits and hippocampus neuron damage. Molecular analysis revealed that GAS exerted the protective effect via reducing the levels of Aβ1–40/42, APP, and β-site APP-cleaving enzyme 1 expression, and increasing Aβ-related protein, a disintegrin and metalloprotease 10, and insulin degrading enzyme expression. Meanwhile, GAS inhibited excessive autophagy via decreasing Beclin-1, LC3-II, and p62 levels. Furthermore, GAS inhibited apoptosis through the downregulation of Bax and upregulation of Bcl-2. Moreover, P38 MAPK signaling pathway was involved in the process. Our findings demonstrate that GAS was effective in the treatment of BCCAO-induced VD via targeting Aβ-related protein formation and inhibiting autophagy and apoptosis of hippocampus neurons.

## Introduction

Vascular dementia (VD), a kind of acquired intelligence damaging syndrome, is characterized by neurodegeneration, cognitive impairment, and memory difficulty. With a considerable growth of elderly population, VD is becoming the second most common type of dementia after Alzheimer’s disease (AD). Unfortunately, up to now, effective therapeutic approaches are unavailable (Smith, 2017). With an urgent demand for novel neuroprotective strategies to treatment of VD, numerous studies have been taken to search effective therapies. Among these studies, bilateral common carotid artery occlusion (BCCAO) in rats is widely accepted as an experimental model, which can imitate the pathological change occurred in VD successfully (Jiwa et al., 2010).

Currently, the underlying mechanisms of VD have been linked to hippocampus neuron damage, inflammatory response, and oxidative stress (Jiwa et al., 2010; Zhang et al., 2018). Furthermore, BCCAO-induced cognitive impairment and the occurrence of VD-like pathogenesis characterized by the deposition of beta-amyloid (Aβ) in the hippocampus are found (Cai et al., 2017), and emerging strategies for interfering with the metabolism of Aβ might be promising therapeutic approaches to attenuate or reverse negative neurological consequences of BCCAO (Won et al., 2013; Zhu et al., 2017). **Autophagy is thought to contribute to** the clearance of Aβ. However, either too much or too little **autophagy** is harmful to neuron. Growing evidence in **support** of abnormal autophagy is correlated with Aβ aggregation after BCCAO. Aβ mediated **autophagy** flux and accumulation of autophagosomes, and the inhibition of **autophagy** decreased Aβ-induced cytotoxicity after BCCAO **injury** (Nagatani et al., 2012; Zou et al., 2017). Simultaneously, other mechanisms such as apoptosis of hippocampal neurons may be also triggered by the accumulation of Aβ (Zhang Y. et al., 2016; Xiong et al., 2017).

Due to the complexity of VD pathological processes, increasing interests have switched to the natural products extracted from herbs, which exert multi-target effects, less adverse drug reactions. *Gastrodia elata* Blume, commonly called Tian ma 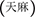 in Chinese, described to enter the liver meridian used for calming liver to stop endogenous Wind. Gastrodin (GAS; **Supplementary Figure S1**) is the principal bioactive component derived from the rhizome of *G. elata*, which has been used as a traditional Chinese medicine to treat cardiovascular diseases, convulsive illness, dizziness, and headache for thousands of years (Jang et al., 2015; Liu et al., 2018). Meanwhile, GAS has received much attention for its various pharmacological activities in the central nervous system, such as anti-neuroinflammation (Zhang J.S. et al., 2016), anti-seizure (Jin et al., 2018), and anti-depression (Zhang et al., 2014). Our previous studies have demonstrated that GAS is able to ameliorate subacute phase of cerebral ischemia-reperfusion injury by inhibiting inflammation and apoptosis (Liu et al., 2016). Previous clinical trials were also revealed that GAS used alone and combination of donepezil was effective in reversing the cognitive impairment in VD patients (Liu et al., 2018). However, its possible mechanisms of GAS on spatial memory impairment of VD have not been elucidated yet.

Therefore, the present study was designed to investigate whether GAS exert beneficial to BCCAO-induced cognitive impairment, and explore its underlying mechanisms, focusing on Aβ-related proteins, and autophagy and apoptosis.

## Materials and Methods

### Drugs

The GAS (C_13_H_18_O_7_; molecular weight: 286.28; purity ≥ 98%) was purchased from Nanjing Zelang Medical Technology Co., Ltd. (Nanjing, China). GAS was dissolved in normal saline (NS). All reagents were of analytical-reagent grade and were generally commercially available.

### Animals

Adult male Sprague-Dawley rats weighing 260 ± 20 g were purchased from the Experimental Animal Center of the Third Military Medical University (SPF grade, Certificate No. SCXK2007-0005). The study protocol was approved by the Experimental Animal Ethics Committee at the Zunyi Medical University. All animals were allowed adaptive feeding for a week prior to experimentation. Rats were housed under a 12 h light/dark cycle at 22–24°C with free access to food and water. Efforts were made to minimize the number of animals tested and their suffering. Animals were randomly divided into the following six groups: Sham, Sham + GAS (60 mg/kg), BCCAO, and BCCAO + GAS (15, 30, and 60 mg/kg) groups, respectively. All animals were orally gavaged with GAS or saline daily at the seventh day after surgery for 28 days.

### Surgery

The BCCAO model in rats was carried out as previously reported (Li et al., 2015; Zhang Y. et al., 2016). Briefly, after deeply anesthetization with 2% sodium pentobarbital (3 ml/kg, i.p.), the bilateral common carotid arteries of the animals were exposed through a midline incision in the neck and carefully separated from the peripheral tissues, then ligated with surgical silk. The operations were performed on a heating pad to maintain body temperature of rats at 37.5 ± 0.5°C, and the rats were kept on the pad until recovery from anesthesia.

### Morris Water Maze

**FIGURE 1:**
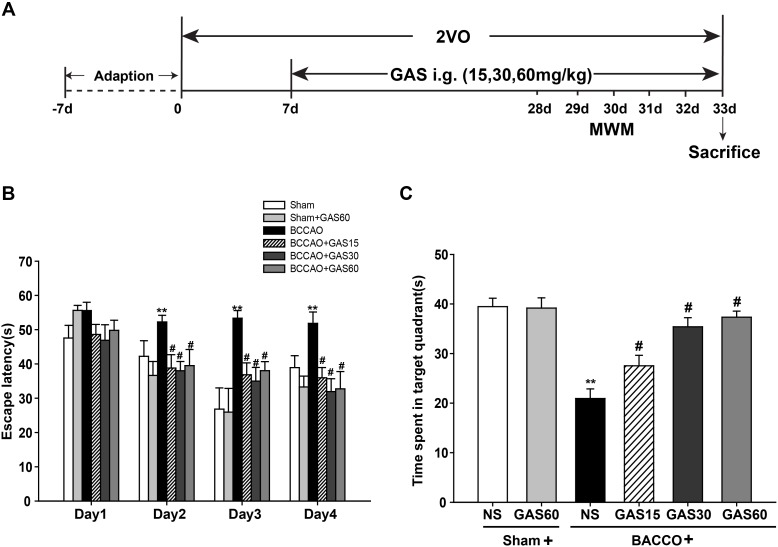
Effects of GAS on Morris water maze performance deficits induced by BCCAO in rats. **(A)** Schematic representation of the experimental design. **(B)** The latencies were measured to assess the rat learning and memory ability in 4 days training trials. **(C)** The percentage of time in the target quadrant. Data are presented as means ± SEM (*n* = 10). ^∗^*P* < 0.05; ^∗∗^*P* < 0.01 vs. sham; ^#^*P* < 0.05; ^##^*P* < 0.01 vs. BCCAO.

Spatial learning and memory of all rats were evaluated using the Morris water maze (MWM) test as described earlier (Hu et al., 2017). Place navigation test was performed from day 24 after operation for 4 consecutive days (**Figure 1A**). In brief, the circular heated water pool was in the diameter 120 cm and height 50 cm, water temperature of 24–26°C was maintained inside, and 1 kg of powdered milk was added to make the water opaque. All external visual clues surrounded by the pool were kept constant for the spatial orientation of the rats. A platform was submerged 2 cm below the water surface in the middle of the northwest quadrant.

Each animal was softly put into water at one of the four starting positions facing the wall of the pool. The swimming time of each rat from the start location to reach the submerged platform (escape latency) was recorded. If the animal missed the hidden platform within the specified time, it was guided to stay on the platform for 15 s and the escape latency was recorded as 120 s. On the fifth day, the platform was removed and the rat was allowed to swim in the pool freely for 120 s, the time and frequency spent in the target quadrant were measured. Performance was taken notes by a computer-based video tracking system and analyzed by the MT-200 image analyzing software (Taimeng Co., Chengdu, China).

### Hematoxylin–Eosin (HE) Staining

Rats were sacrificed after the MWM, and brains were perfused by a tip transfusion pump through which inserted from apex area of heart into the aorta perfusion, perfused with 0.9% cold phosphate buffered saline and 4% paraformaldehyde. After that, the brain tissues were removed and 4% paraformaldehyde was used to incubate for 24 h at 4°C. Brain samples behind optic chiasm including the whole hippocampus were removed to embed in paraffin. Embedded brain tissue sections were continuous sliced into 4–5 μm sections for histological analysis according to the instructions. Light microscopy was used to observe the histomorphology of neurons, and CA1 and CA3 subfields of hippocampi counted at 400× magnification were selected to check for morphological alterations.

### Flow Cytometry With AnnexinV-FITC/PI Double Staining

Flow cytometry analysis of tissue was performed as described previously (Schutte et al., 1998; Erisgin and Tekelioglu, 2017; Kim et al., 2017). Annexing-FITC/PI double staining was performed and the apoptosis rate of hippocampi was tested with flow cytometry. Hippocampal tissue was added with 500 μL physiological saline, grinded with a glass rod, and the cell suspension was filtered through a 200-mesh sieve. Washed with pre-cooled PBS for three times, cells were centrifuged for removal of the supernatant. Cells were re-suspended with 500 μL blinding buffer, reacted with 5 μL annexin V-FITC and 5 μL PI at room temperature for 5–10 min in the dark, and then quantified using flow cytometry (Beckman Coulter, United States) at an excitation wavelength of 480 nm and an emission wavelength of 530 nm.

### Western Blot Analysis

After rats were sacrificed, the isolated hippocampal tissues were used for Western blot analysis. The tissues were homogenized in ice-cold lysis buffer supplemented with protease inhibitors and phosphatase inhibitors. The tissues were centrifuged at 4°C for 15 min, and supernatant was collected. Protein concentration was measured using the BCA assay kit. Then, the lysates were separated with 5–12% SDS–PAGE and transferred to PVDF membranes. Followed by electrophoresis, PVDF membranes were blocked by 5% non-fat milk at room temperature. After that, membranes were incubated overnight at 4°C with primary antibodies against the following proteins: Aβ 1-40 (Aβ_1-40_, 1:1000, Abcam, United States), Aβ 1-42 (Aβ_1-42,_ 1:1000, Abcam, United States), amyloid precursor protein (APP, 1:1000, Sangon Biotech, China), β-site APP-cleaving enzyme 1 (BACE1, 1:1000, Sangon Biotech, China), a disintegrin and metalloprotease 10 (ADAM10, 1:1000, Abcam, United States), insulin degrading enzyme (IDE, 1:1000, Abcam, United States), autophagy-related protein Beclin-1 (Beclin-1, 1:1000, Abcam, United States), autophagy marker Light Chain 3 (LC3, 1:1000, Novus Biologicals, United States), nucleoporin p62 (p62, 1:1000, Abcam, United States), phosphorylation of P38 mitogen-activated protein kinase (p-P38MAPK, 1:2000, Abcam, United States), P38 mitogen-activated protein kinase (P38MAPK, 1:2000, Abcam, United States), B cell lymphoma/leukemia-2 (Bcl-2, 1:2000, Abcam, United States), and Bcl-2-associated X protein (Bax, 1:2000, Abcam, United States). Then, the membranes were incubated with the relevant secondary antibodies for 2 h. Blots were visualized using chemiluminescence reagent BeyoECL Plus (Beyotime). Quantity One 1-D analysis software v4.52 (BioRad) was used for scanning the image and quantifying band intensity.

### Statistical Analysis

Repeated measures multivariate analysis of variance was used to assess the results of MWM, others were analyzed using one-way analysis of variance (ANOVA) with SPSS 17 (SPSS, United States). A value of *P* < 0.05 was considered statistically significant.

## Results

### GAS Attenuates Learning and Memory Impairments in the BCCAO Rats

The MWM was used to evaluate the spatial learning and memory function of GAS treatment. A training trial lasts for 4 days, and the escape latency time was recorded. As shown in **Figure 1B**, the animals in BCCAO group presented significantly prolonged escape latency than that of sham group (*P* < 0.01), indicating the impairment of the learning and memory performances representing in VD models. However, treatment with GAS significantly mitigated the poor spatial learning in the MWM test (*P* < 0.05). We also found Sham and Sham + GAS (60 mg/kg) did not show any change on learning and memory impairments. Subsequently on the fifth day, we performed a probe trial to evaluate the spatial memory retention among different groups. Results showed that rats in BCCAO group presented worse memory ability with spent less time in the platform quadrant than that of Sham and Sham + GAS (60 mg/kg) groups (*P* < 0.05). However, GAS (15, 30, and 60 mg/kg, respectively) treatments significantly arrested the spatial memory impairment (**Figure 1C**, *P* < 0.05).

### GAS Attenuates Neuronal Damage in the Hippocampus Induced by BCCAO

**FIGURE 2:**
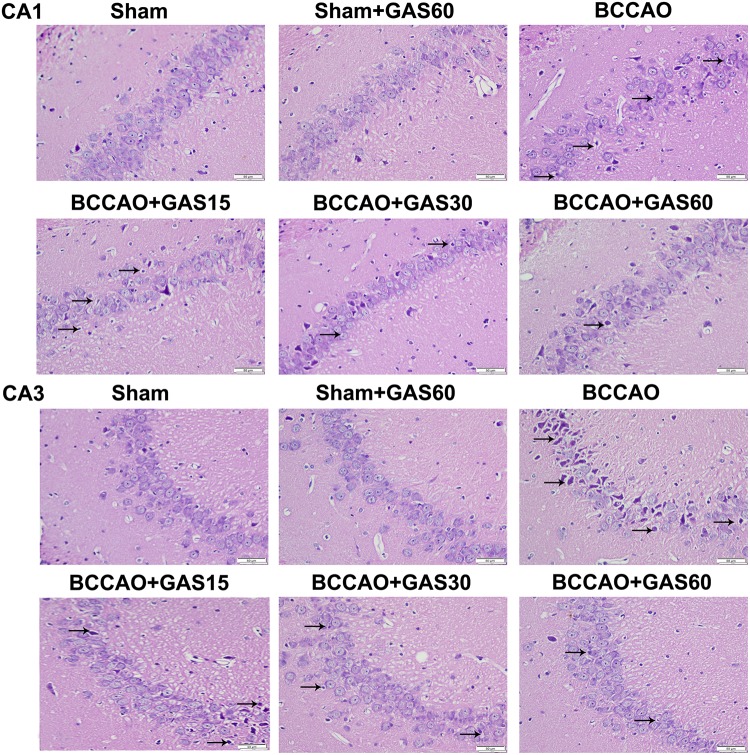
Effects of GAS on BCCAO-induced morphological alterations in the hippocampi CA1 and CA3 subfields after BCCAO (Magnification 400×, Scale bar = 50 μm). Representative sections were stained using HE. Normal cellular morphology was present in the Sham and Sham + GAS (60 mg/kg) groups. Compared with the BCCAO group, a gradual improvement in condensed nuclei (arrows) was detected in the hippocampal CA1 and CA3 region in each GAS treatment group.

The rat hippocampal CA1 and CA3 region was observed by HE staining (**Figure 2**). The results showed that most neurons were lost, shrinkage, dark-stained, and with severe cellular edema both in the CA1 and in the CA3 areas of the hippocampus in BCCAO group compared with Sham and Sham + GAS (60 mg/kg) groups. However, GAS-treatment groups attenuated BCCAO-induced neuronal damage, especially in the GAS (60 mg/kg) group.

### GAS Suppresses Apoptosis in the BCCAO Rats

**FIGURE 3:**
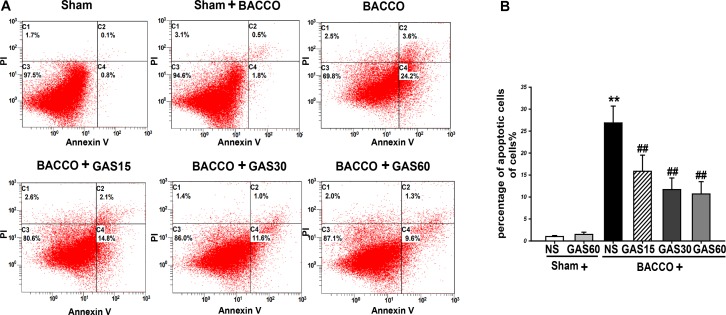
Effects of GAS on BCCAO-induced apoptosis in the hippocampus of rats. **(A)** The apoptosis cells were measured by flow cytometry. Each image has four quadrants: C1, necrotic cells; C2 and C4, apoptotic cells; C3, viable cells. **(B)** Quantitative analysis of apoptosis. Data are presented as means ± SD (*n* = 3). ^∗^*P* < 0.05; ^∗∗^*P* < 0.01 vs. sham; ^#^*P* < 0.05; ^##^*P* < 0.01 vs. BCCAO.

Analysis of BCCAO induced apoptosis in the hippocampus using Annexing-FITC/PI double staining assay, and the rate of apoptosis cells in the hippocampus of BCCAO was to determine the addition percentages of C2 (late apoptotic and necrotic cells) and C4 (early apoptotic cells). As is shown in **Figure 3**, BCCAO group was increased compared with Sham and Sham + GAS (60 mg/kg) groups (*P* < 0.01). GAS was able to inhibit the BCCAO-induced cell apoptosis in a dose-dependent manner (*P* < 0.01).

### GAS Reduces Aβ_1-40_ and Aβ_1-42_ Level in the Hippocampus Induced by BCCAO

**FIGURE 4:**
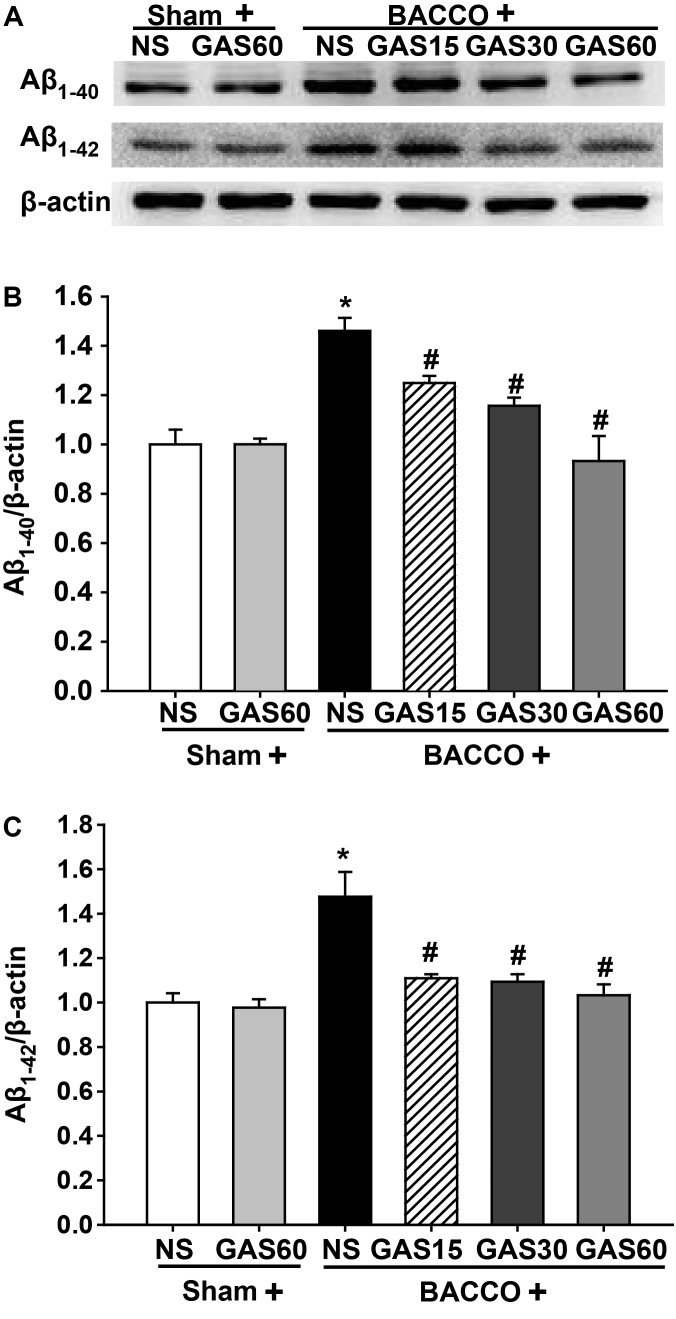
Effects of GAS on Aβ_1-40_ and Aβ_1-42_ protein level in the hippocampus. **(A)** Aβ_1-40_ and Aβ_1-42_ protein level in the hippocampus. **(B,C)** Quantitation of Aβ_1-40_ and Aβ_1-42_ levels. Values are mean ± SD (*n* = 3). ^∗^*P* < 0.05; ^∗∗^*P* < 0.01 vs. sham; ^#^*P* < 0.05; ^##^*P* < 0.01 vs. BCCAO.

Western blot results (**Figure 4**) showed that a significant increase in the level of Aβ_1-40/42_ protein in the group of BCCAO rats (*P* < 0.05), as compared to those treated with or without GAS. However, when groups were administered with different concentrations of GAS simultaneously, the BCCAO-induced Aβ_1-40/42_ protein increase could be considerably prevented (*P* < 0.05).

### GAS Regulates the Expression of Aβ-Related Protein in the Hippocampus of the BCCAO Rats

**FIGURE 5:**
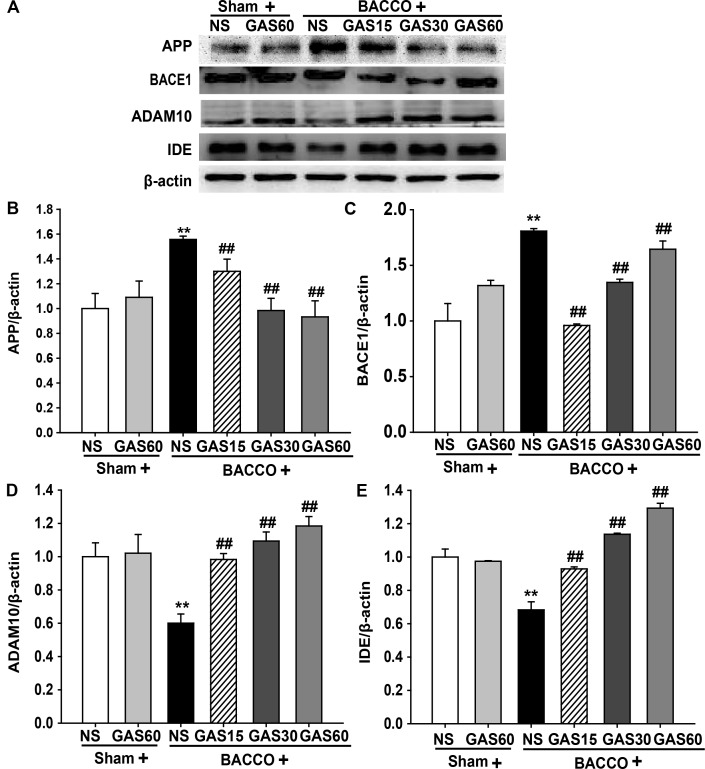
Effects of GAS on Aβ-related protein expression in the hippocampus. **(A)** APP, BACE1, ADAM10, and IDE protein expression in the hippocampus. **(B–E)** Quantitation of APP, BACE1, ADAM10, and IDE levels, respectively. Values are mean ± SD (*n* = 3). ^∗^*P* < 0.05; ^∗∗^*P* < 0.01 vs. sham; ^#^*P* < 0.05; ^##^*P* < 0.01 vs. BCCAO.

Further to explore the mechanisms of GAS on reduction of BCCAO-induced deposition of Aβ, the level of Aβ-related protein were detected using Western blot (**Figure 5**). The expressions of APP and BACE1 were significantly increased in BCCAO group compared with Sham and Sham + GAS (60 mg/kg) groups (*P* < 0.01). However, BCCAO-induced high expression of APP and BACE1 has been prevented by various doses of GAS. Meanwhile, the low expression of ADAM10 and IDE in BCCAO group was also significantly raised by GAS in different doses (*P* < 0.01).

### GAS Regulates the Expression of Autophagy-Related Protein in the Hippocampus of the BCCAO Rats

**FIGURE 6:**
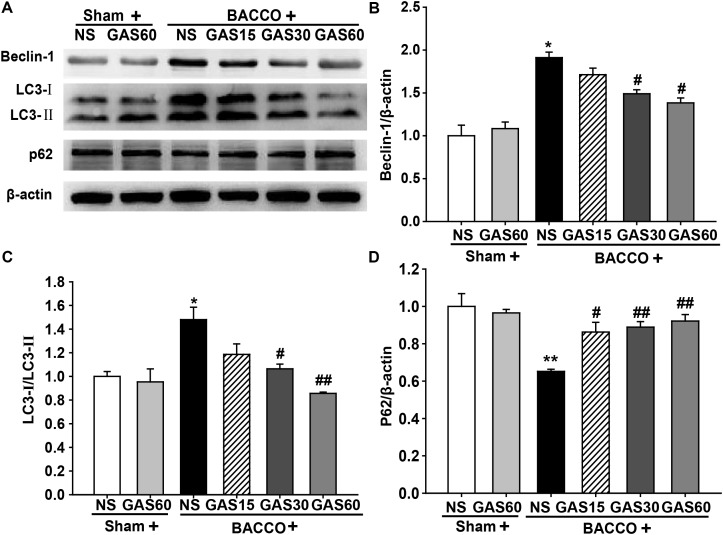
Effects of GAS on autophagy-related protein expression in the hippocampus. **(A)** Beclin-1, LC3–II, and p62 protein expression in the hippocampus. **(B–D)** Quantitation of Beclin-1, LC3–II, and p62 levels, respectively. Values are mean ± SD (*n* = 3). ^∗^*P* < 0.05; ^∗∗^*P* < 0.01 vs. sham; ^#^*P* < 0.05; ^##^*P* < 0.01 vs. BCCAO.

To further confirm the effect of GAS on abnormal autophagy, we examined autophagy-related proteins in each group by Western blot (**Figure 6**). Beclin-1 and LC3-II were increased, simultaneously, p62 was decreased in the BCCAO group compared with that in Sham and Sham + GAS (60 mg/kg) group, but these effects were reversed by treatment with various doses of GAS (*P* < 0.05).

### GAS Regulates the Expression of Apoptosis-Related Protein in the Hippocampus of the BCCAO Rats

**FIGURE 7:**
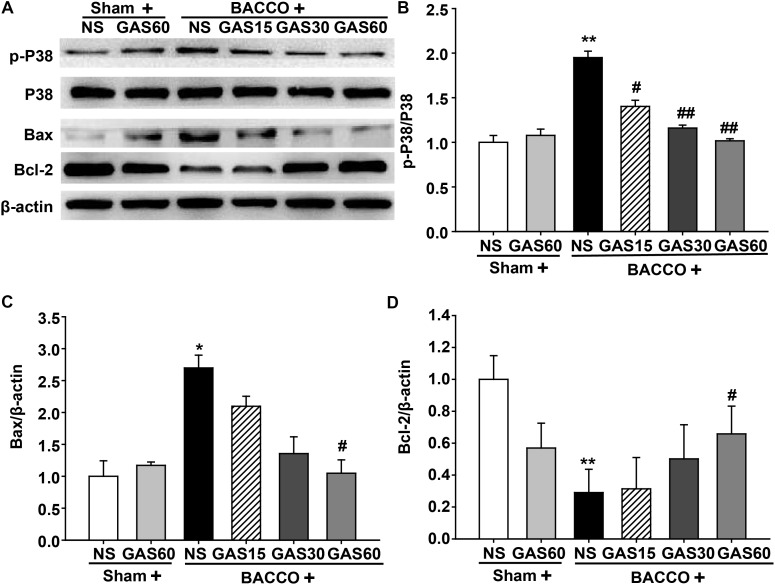
Effects of GAS on apoptosis-related protein expression in the hippocampus. **(A)** P38 MAPK, Bax, and Bcl-2 protein expression and P38 MAPK phosphorylation in the hippocampus. **(B–D)** Quantitation of p-P38 MAPK, P38 MAPK, Bax, and Bcl-2 levels, respectively. Values are mean ± SD (*n* = 3). ^∗^*P* < 0.05; ^∗∗^*P* < 0.01 vs. sham; ^#^*P* < 0.05; ^##^*P* < 0.01 vs. BCCAO.

As shown in **Figure 7**, BCCAO resulted in a significantly increase of p-P38 MAPK and Bax and a drastic decrease of Bcl-2, but had no change on total P38 MAPK. As expected, increased p-P38 MAPK and Bax were rescued by GAS in a dose-dependent manner. Meanwhile, GAS was able to increase Bcl-2 expression.

## Discussion

The present study revealed that GAS treatment effectively attenuated BCCAO-induced cognitive deficits, reduced hippocampus CA1 and CA3 pyramidal neuron morphological damage. Molecular biology analysis revealed that its underlying mechanism was mediated, at least in part, by attenuating deposition of Aβ, reducing BCCAO-enhanced autophagy, and suppressing neurons apoptosis. These novel findings provide pharmacological basis of GAS for VD.

Our previous studies found that rats subjected to BCCAO showed a significant decrease in hippocampus-dependent cognitive impairment (Li et al., 2015). Consistent with this observation, the present results of MWM demonstrated that significant learning and memory impairments appeared in BCCAO rats. However, long-term treatment with GAS significantly arrested the cognitive impairment. Furthermore, HE staining revealed that morphological defects in the CA1 and CA3 areas of hippocampus were also founded, and GAS treatment ameliorated morphological damage in the CA1 and CA3 region, which was consistent with the behavioral results of the MWM. These results clearly demonstrate that GAS has beneficial effects on the cognitive impairment in rats with BCCAO.

A great quantity of Aβ deposition has been reported in BCCAO rats, and the degree of cognitive impairment is positively correlated with the expression of Aβ (Li et al., 2015). Thus, the strategies of restrain Aβ production and/or promote Aβ clearance can reduce the levels of Aβ, and can set-back BCCAO-induced cognitive impairment (Choi et al., 2011; Song et al., 2013; Han et al., 2015; Reijmer et al., 2016; Cai et al., 2017). Moreover, Aβ_1-40_ and Aβ_1-42_ are the two most pre-dominant forms of Aβ in the plaques. Previous research has suggested that GAS treatment markedly reduced the level of Aβ_1-40_ and Aβ_1-42_ and reversed cognitive deficit in Aβ_1-42_-injected mice (Li and Qian, 2016). Therefore, the levels of Aβ_1-40_ and Aβ_1-42_ in the hippocampus were detected. Similar to prior researches, the contents of Aβ_1-40_ and Aβ_1-42_ were significantly increased after BCCAO. However, GAS-treated BCCAO rats shown a reduction in Aβ_1-40_ and Aβ_1-42_ level. Therefore, one of the molecular mechanisms by which GAS attenuates BCCAO-induced cognitive deficits is related to elimination of Aβ.

As is known that, APP, the raw material of generation of Aβ, is processed along two alternative pathways: one is amyloidogenic pathway, in which β- and γ-secretases lead to the accumulation of Aβ, activation of β-secretase could generate neurotoxic Aβ; the other pathways is non-amyloidogenic pathway, which α- and γ-secretases lead to the production of soluble amyloid precursor protein-α (sAPPα). It is completely different from the amyloidogenic pathway, and the production of non-amyloidogenic pathway, sAPPα, has neurotropic and neuroprotective properties (Meineck et al., 2016). There is wide consensus that activation of α-secretase could increase production of neuroprotective sAPPα (Zhang et al., 2012; Corrigan et al., 2012a,b; Jeong, 2017; Yuksel et al., 2017). In productive process of Aβ, BACE1 is responsible for the chief function of β-secretase (Maloney and Lahiri, 2011). Once BACE1 unregulated, APP is processed along the amyloidogenic pathway, and resulted in the overexpression of neurotoxic Aβ (Zhu et al., 2013). ADAM10, a competitor of BACE1, is not only promoting the expression of α-secretase that combats formation of amyloidosis, but also enhancing release of neuroprotective sAPPα. Furthermore, in clean-up process of Aβ, IDE is one of the major proteases responsible for Aβ clearance enzyme in the hippocampal lysates and for the degradation of Aβ in cerebrospinal fluid and cytoplasm (Stargardt et al., 2013; Kukreja et al., 2014). Interestingly, in this study, we found that GAS not only inhibited Aβ production, but also promoted Aβ clearance following BCCAO in rats. Altogether, GAS downregulated BACE1, indirectly decreased Aβ neurotoxic injury, which re-confirm previous findings (Zhang J.S. et al., 2016). The findings that GAS upregulated ADAM10 and IDE expression are novel, and may be important against Aβ etiology.

The aggregation of Aβ after BCCAO is also correlated with abnormal autophagy. Aβ mediated autophagy flux and accumulation of autophagosomes; meanwhile, Aβ deposition was found to induce neuron apoptosis (Ghavami et al., 2014). Thus, novel agents targeting apoptosis inhibition and/or autophagy regulation might be potential neuroprotective drugs for VD. Previous studies have suggested that GAS treatment regulates autophagy and apoptosis dysfunction in astrocytes exposed to LPS *in vitro* (Wang et al., 2016), however, the effect of GAS on VD has not been reported. Here, we focused on the expression of autophagy and apoptosis mediators implicated in VD pathology. Autophagy, a double-edged sword, can be activated in different stress responses and in many pathological processes of diseases, which is thought to be a protective mechanism. Nevertheless, excessive autophagic activity may lead to a collapse of cellular functions. In this study, we detected some markers of autophagy, such as Beclin-1, LC3-II, and p62. Beclin-1 is an important autophagy-regulatory gene that reflects autophagic activity (Shin et al., 2014). Besides, LC3-II embedded on the membrane of autophagic vacuole, the content of which represents autophagy activity. p62, an autophagy regulatory factor, overexpression of p62 promotes the degradations of abnormal proteins (Xu et al., 2014). Consistent with previous findings, we found that Beclin-1, LC3-II, and p62 were increased after BCCAO (Gao et al., 2015; Zou et al., 2017). Fortunately, GAS treatment markedly decreased the expression of Beclin-1, LC3-II, and p62, indicating BCCAO activated autophagic flux, and GAS repressed BCCAO-induced autophagic flux. Moreover, we found autophagy suppression may not be the unique mechanism of the protective effect of GAS on BCCAO-induced injury, and apoptosis inhibition may also be involved. Apoptosis plays an important role in the process of neuronal death after BCCAO (Zhao et al., 2018). As a result of GAS treatment, a marked reduction in the number of apoptotic cells in the hippocampus was observed (**Figure 3**). Further study indicated that GAS against apoptosis via enhancing Bcl-2 expression and reducing Bax expression. It is worth mentioning that, Bcl-2 is not only an anti-apoptotic protein, but also an anti-autophagy protein via interaction with Beclin-1(Mukhopadhyay et al., 2014). Thus, we therefore postulated that the protective effect of GAS on VD is associated with upregulation of Bcl-2, and then combined with excessive Beclin-1 to block autophagy. These findings indicated that attenuation of excessive autophagy and apoptosis is important molecular mechanisms of GAS against VD.

The MAPK consists of three well-defined subgroups, among which P38 MAPK is closely associated with autophagy and apoptosis (Ghatan et al., 2000; Jiang et al., 2013; Han et al., 2014; Xue et al., 2015). Coincidentally, GAS showed inhibitory effect on P38 MAPK phosphorylation in previous studies (Yang et al., 2013; Jiang et al., 2014), thus we assessed the phosphorylation of P38 MAPK. As expected, treatment with GAS inhibited phosphorylation of P38 MAPK. Thus, we speculate that GAS blocks P38 MAPK activation, and consequently suppress excessive autophagy and apoptosis. Regretfully, downstream of P38 MAPK signaling pathway, related to autophagy and apoptosis, has not been rigorously studied in this study, and selective P38 MAPK antagonists or P38 MAPK knock-out models will be further required to clarify the exact molecular mechanism. Thus, the beneficial effects of GSA also involve its modulation on MAPK signaling pathway.

## Conclusion

We demonstrated that GAS attenuates BCCAO-induced cognitive deficits and hippocampus neuron damage, and its underlying mechanism is likely due to decreasing Aβ deposition, retraining excessive autophagy and apoptosis, and regulating P38 MAPK signaling pathway.

## Author Contributions

J-SS conceived and designed all the experiments. BL, J-MG, and FL performed the experiments. FL and Q-HG finished the data analysis. BL, J-MG, FL, and Q-HG wrote and revised the manuscript. All the authors reviewed the manuscript and approved the submitted manuscript.

## Conflict of Interest Statement

The authors declare that the research was conducted in the absence of any commercial or financial relationships that could be construed as a potential conflict of interest.

## Supplementary Material

FIGURE S1 Chemical structure of gastrodin.
