# Supplementary figures and images for: Gastrodin Attenuates Bilateral Common Carotid Artery Occlusion-Induced Cognitive Deficits via Regulating Aβ-Related Proteins and Reducing Autophagy and Apoptosis in Rats
Source: Front Pharmacol. 2018 Apr 26;9:405. doi: 10.3389/fphar.2018.00405 (PMC5932202; doi:10.3389/fphar.2018.00405)

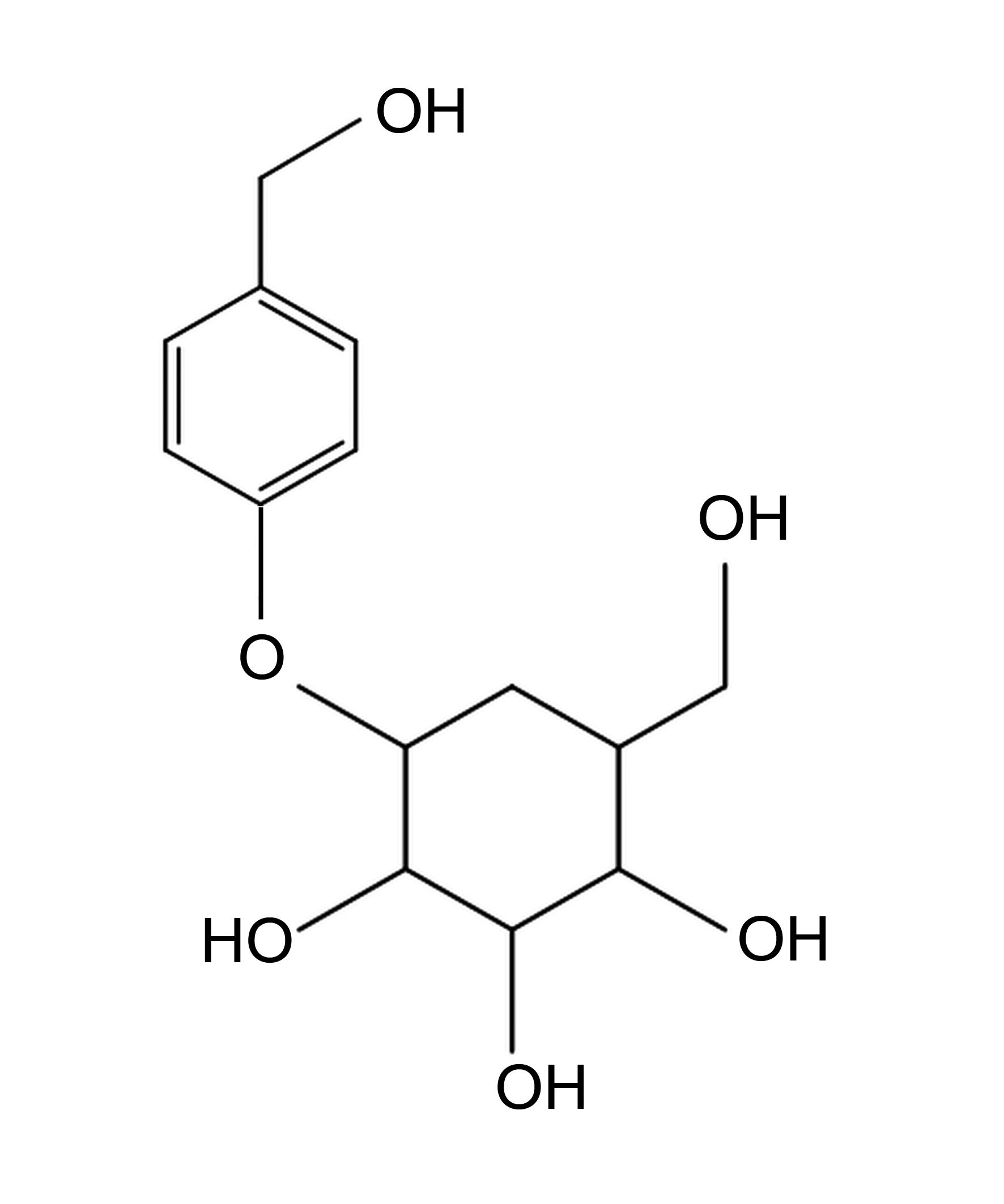

Supplement: FIGURE S1 — Chemical structure of gastrodin. [file Image_1.TIF]
